# Development of an immune-related prognostic index associated with osteosarcoma

**DOI:** 10.1080/21655979.2020.1864096

**Published:** 2020-12-29

**Authors:** Chao-Dong Yin, Ying-Lan Hou, Xiao-Ren Liu, Yu-Sheng He, Xin-Ping Wang, Cheng-Jie Li, Xiao-Hong Tan, Jun Liu

**Affiliations:** a Department of Hand and Foot Surgery and Microsurgery, Affiliated to the First People’s Hospital of Chenzhou, P.R. China; b Health Management Centre, Affiliated to the First People’s Hospital of Chenzhou, P.R. China

**Keywords:** Osteosarcoma, tumor immunity, prognostic index

## Abstract

Tumor immunity is closely associated with the prognosis of tumors, including osteosarcoma (OS). The aim of the present study was to construct an immune-related prognostic index (PI) to predict the prognosis of OS. Herein, OS expression data were sourced from the Therapeutically Applicable Research to Generate Effective Treatments (TARGET) *database*. We divided the OS patients into nonmetastatic and metastatic groups, allowing differentially immune-related genes (DIRGs) to be selected. After DIRGs were further investigated by enrichment analysis, four keys prognostic IRGs (CD79A, CSF3R, MTNR1B and NPPC) *were identified using a Cox proportional hazards model*. Then, an immune-related prognostic index was constructed. Finally, gene set enrichment analysis (GSEA) was employed to further explore the underlying mechanisms. The difference in tumor-infiltrating immune cell (TIIC) abundance was also discussed. In our study, eight upregulated genes and 30 downregulated genes were identified. Several Gene Ontology (GO) terms and the most significantly enriched KEGG pathways were immune-associated functions and pathways. Four genes, including CD79A, CSF3R, MTNR1B and NPPC, were used to establish a risk assessment model for evaluating OS prognosis. *GSEA revealed that the risk score was related to cytokine receptor interaction and to the chemokine and B cell receptor signaling pathways. Furthermore, high risk markedly related to the infiltration of several immune cell types, including M2 macrophages, naïve CD4 T cells, and CD8 T cells*. In sum, we developed a survival model for OS. The underlying molecular mechanisms of the high-risk group may affect immune-related biological processes and TIICs.**Abbreviations TARGET**: Therapeutically Applicable Research To Generate Effective Treatments; PI: Prognostic index; OS: Osteosarcoma; DIRGs: Differentially immune-related genes; GSEA: Gene set enrichment analysis; TIIC: Tumor-infiltrating immune cell.

## Graphical abstract

**Figure:**
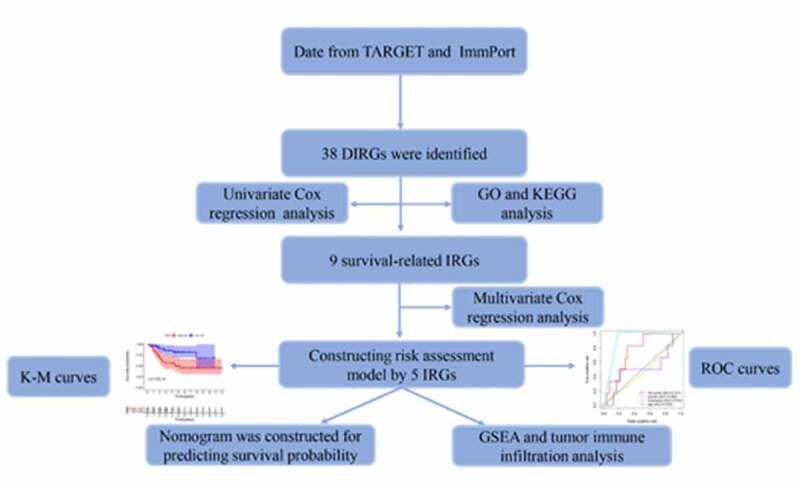

## Introduce

Osteosarcoma (OS) is a primary malignant bone tumor with a morbidity of 4,000,000 annually in children and adolescents [1,2]. Despite advances in surgery, chemotherapy and radiotherapy, the cumulative 5-year overall survival rate for OS patients without metastasis is 77.9% [3]. However, OS still has a mortality rate of 30% [4]. Immunosuppressive therapies have significantly improved the prognosis of OS patients, which provides a reference for further study on the association between abnormal immune gene expression and OS prognosis [5,6].

Several studies have revealed that tumor immunity is closely associated with metastasis and chemoresistance in OS [7–9]. For instance, a microenvironment with low CD4+ and CD8+ tumor infiltrating lymphocytes (TILs) may contribute to a weakened antitumor immune response [10]. Programmed cell death receptor-ligand 1 (PD-L1) is a glycoprotein expressed on the cell surface of T and B lymphocytes, dendritic cells, macrophages, and tumor cells [11,12]. PD-L1 has been shown to modulate drug resistance to paclitaxel and doxorubicin and OS cell growth [50% and 23.7% intermediate and high expression of OS, respectively] [13]. Importantly, PD-L1 expression in tumor cells is closely associated with the TIL level, which often has a higher risk of tumor metastasis and poorer prognosis [14]. These findings indicate that tumor immunity is a key mediator of tumor migration. Therefore, it is pivotal to study the prognostic value and the clinical relevance of IRGs in OS.

For the current study, original mRNA microarray datasets were downloaded from the TARGET database (65 OS nonmetastatic samples and 22 metastatic OS samples). R software was used to identify DIRGs between nonmetastatic and metastatic samples. The functional roles of DIRGs were examined. Thereafter, the prognostic index (PI), as an independent index for OS prognosis, was developed based on IRGs. The potential mechanisms are also discussed.

## Materials & methods

### Data sources

The RNAseq data and corresponding clinical follow-up information were downloaded from the public TARGET database, which included a total of sixty-five OS nonmetastatic samples and a total of twenty-two OS metastatic samples.

### IRG extraction and DIRG analysis

A total of 2498 genes from the ImmPort Shared Data were identified as IRGs. The R statistical software package ‘limma’ was applied to estimate DEGs. |log(FC)| ≥ 0.5 and p-value< 0.05 were used as cutoffs to identify DIRGs. Using the ‘ggpubr’ and ‘pheatmap’ packages generated boxplots and heatmaps, respectively.

### Function enrichment analysis of DIRGs

The Database for Annotation, Visualization, and Integrated Discovery (DAVID) 6.8 (https://david.ncifcrf.gov) was used for gene function analysis [4]. GO and KEGG pathways were analyzed and visualized by the clusterProfiler R package [15]. The critical value of significance for the GSEA screening was set at P < 0.05.

### Survival analysis

Univariate Cox regression (UCR) analysis was used to identify genes whose expression was related to the overall survival of patients with OS. Genes with a P‐value less than 0.05 were considered candidate genes related to patient survival. The independent prognostic factors were identified by multivariate Cox proportional hazards regression (MCR) analysis. Overall survival was analyzed using the Kaplan-Meier survival curve to assess differences in survival. The specificity for diagnostic accuracy was proven by the receiver operating characteristic (ROC) curve (AUC). We used the cBioPortal tool to determine survival-associated IRG alterations in sarcoma.

### Development of the nomogram

The nomogram was constructed based on metastasis and risk score using the ‘survival’and ‘rms’package for R.

### GSEA

GSEA has been extensively applied to identify underlying pathways. Eighty-seven OS patients were divided into two groups according to the median risk score, including high‐and low‐risk groups. The C2.cp.kegg.v7.0.symbols.gmt dataset was obtained from the Molecular Signatures Database (MsigDB). NOM P-value <0.05, |NES| >1 and FDR q < 0.25 were considered statistically significant.

### CIBERSORT estimation

CIBERSORT provides an abundance ratio estimate using gene expression data of numerous cell types in a mixed cell population [16,17]. The CIBERSORT algorithm was used to analyze an entire data set of twenty-two immune cell subtypes of OS from TARGET, which was grouped into two risk groups (high – and low-risk groups).

### Statistical analysis

All statistical analyses were conducted with RStudio software and SPSS v.23.0 software. Student’s *t*-test for independent samples was carried out to assess the notable differences between both groups. Independent prognostic factors were evaluated using UCR and MCR analysis. P < 0.05 was considered statistically significant.

## Results

### Identification of DIRGs

**Figure 1.**
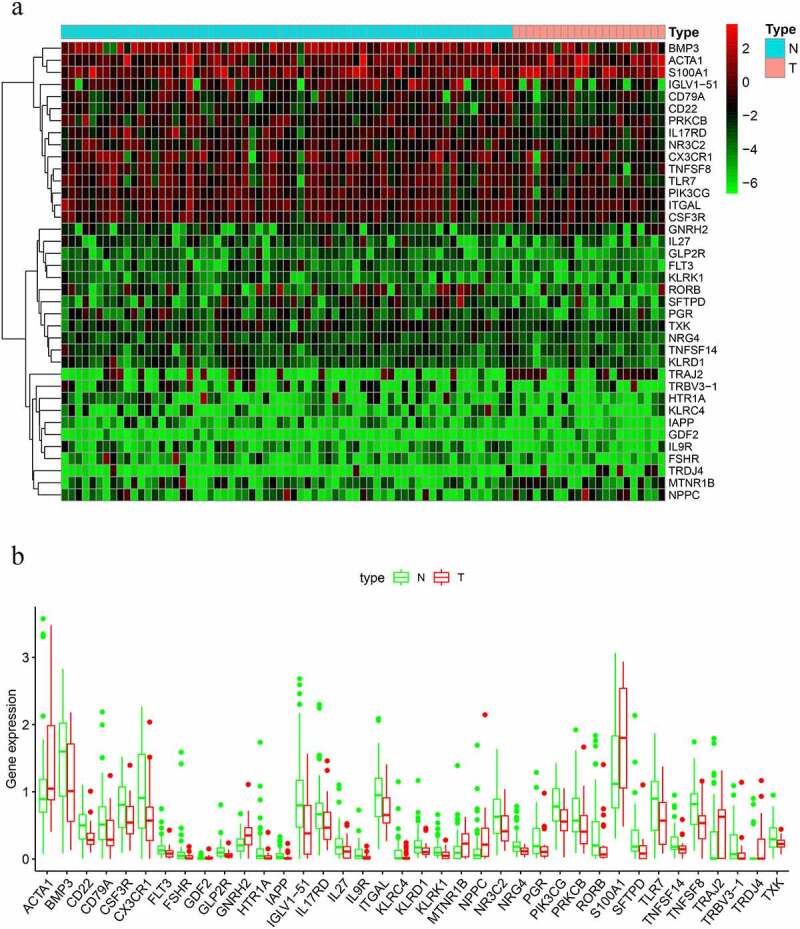
Differentially expressed immune-related genes (IRGs). (a) Heat map. (b) Expression patterns of 29 immune-related genes (IRGs) in nonmetastatic and metastatic osteosarcoma samples. The red dots on the X-axis indicates the metastatic samples and the blue dots indicate the nonmetastatic samples. N, nonmetastatic samples; T, metastatic samples

We identified a total of thirty-eight DIRGs when comparing metastatic samples to nonmetastatic primary samples from the TARGET dataset; eight of these genes were notably upregulated and thirty genes were markedly downregulated (Figure 1).

### DIRG enrichment analysis

**Figure 2.**
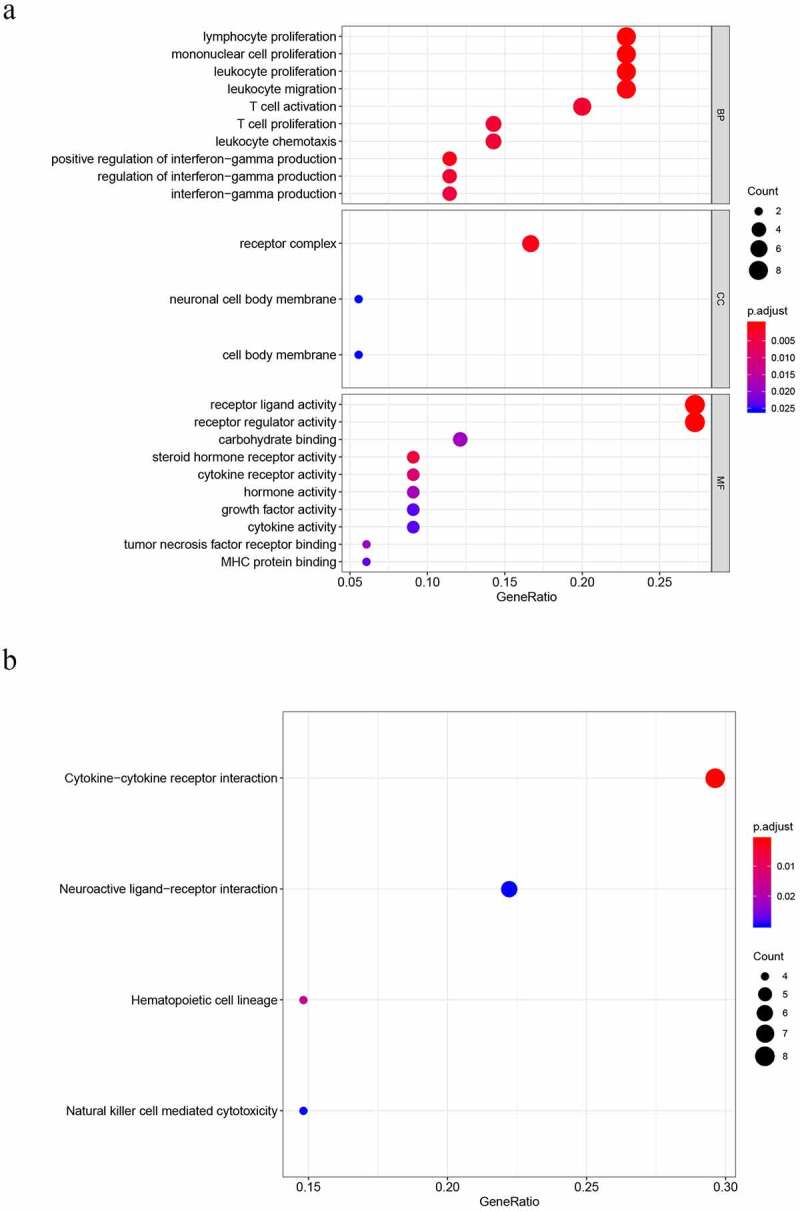
Results of gene functional enrichment. (a) GO analysis shows the biological processes and molecular functions involved in DIRGs. (b) KEGG pathway analysis of differentially immune-related genes

To properly understand the underlying molecular functions (MFs), biological processes (BPs), KEGG pathways, and cellular components (CCs) associated with DEGs, we applied KEGG and GO pathway enrichment analyses. The top 5 terms for BP were as follows: lymphocyte proliferation, mononuclear cell proliferation, leukocyte proliferation, leukocyte migration and T cell activation. The terms of CC were as follows: receptor complex, neuronal cell body membrane, and cell body membrane. The top 5 terms for MF were as follows: receptor ligand activity, receptor regulator activity, carbohydrate binding, steroid hormone receptor activity and cytokine receptor activity (Figure 2(a)). Four significantly enriched signaling pathways were identified, including NK cell-mediated cytotoxicity, hematopoietic cell lineage, neuroactive ligand, and cytokine−cytokine receptor interaction (Figure 2(b)).

### Identification of survival-associated IRGs

**Figure 3.**
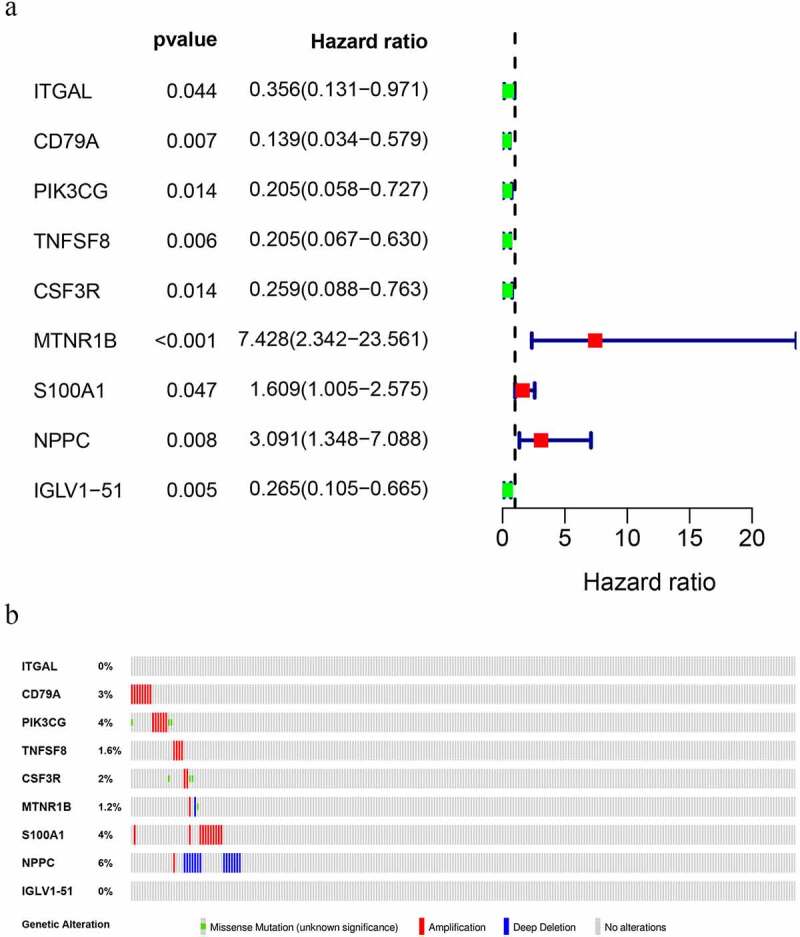
Prognostic value and mutations of IRGs. (a) Forest plot of immune genes related to OS survival. (b) Mutations in prognosis-related IRGs

A total of nine DIRGs related to clinical outcomes were extracted (P < 0.05) (Table 1). Figure 3(a) shows a forest plot of hazard ratios (Fp/HR), illustrating the associations between 9 survival-related IRGs and the overall survival rate in OS patients. The Fp/HR indicated that most IRGs were correlated with better clinical outcomes in OS patients. The mutations of 9 survival-related IRGs were analyzed using cBioPortal, which indicated that several genes had amplification, missense, and deep deletion (Figure 3(b)).

**Table 1.** The specific information of IRGs

ID	Description Name	Expression
ITGAL	Integrin, alpha L (antigen CD11A (p180))	Down
CD79A	CD79a molecule, immunoglobulin-associated alpha	Down
PIK3CG	Phosphoinositide-3-kinase, catalytic, gamma polypeptide	Down
TNFSF8	Tumor necrosis factor (ligand) superfamily, member 8	Down
CSF3R	Colony stimulating factor 3 receptor (granulocyte)	Down
MTNR1B	Melatonin receptor 1B	UP
S100A1	S100 calcium binding protein A1	UP
NPPC	Natriuretic peptide precursor C	UP
IGLV1-51	Immunoglobulin lambda variable 1–51	Down

### Construction of models for prognosis

**Figure 4.**
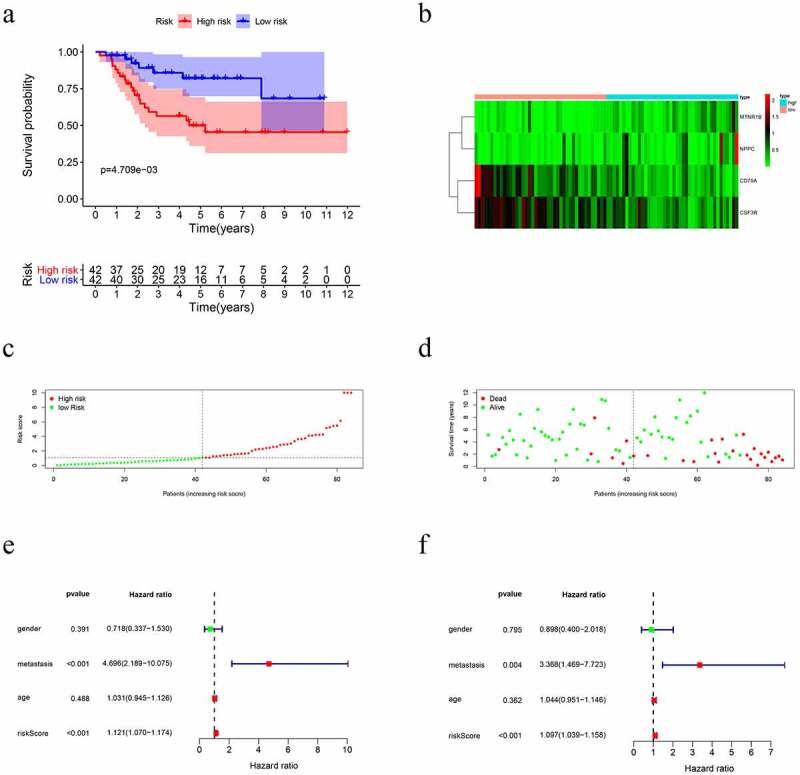
Immune-related prognostic index (PI) of OS patients. (a) Patient overall survival was notably lower in the high-risk group. (b) The heatmap of the four signature genes expression profiles. (c-d) The distribution of risk scores, patient survival time, and status for OS. (e-f) Univariate and multiple regression analysis of OS and the relationships between risk score, sex, metastasis, and age

**Figure 5.**
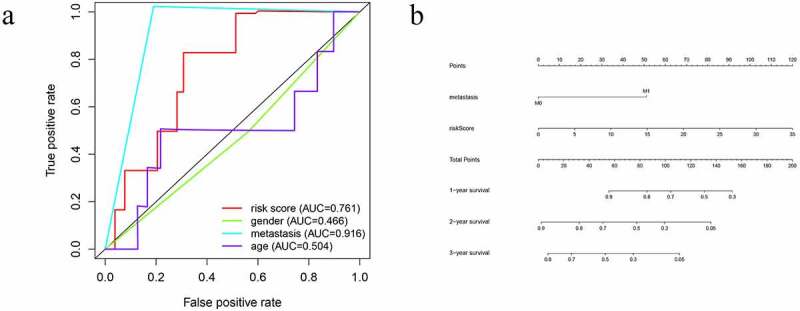
The prognostic value of immune-related prognostic index of OS patients. (a) Survival-dependent receiver operating characteristic (ROC) curves for validation of prognostic value of the prognostic index. (b) The nomogram for predicting overall survival

A PI was developed based on MCR analytical results. Next, we divided the subjects into two cohorts and constructed a risk curve (Figure 4(a–f)). Patients with a low risk score had a higher survival rate than patients with a high risk score. This immune-based PI could be an essential prognostic classification system for patients with OS (Figure 4(d)). The formula for the risk score of every OS patient was constructed: risk score = [CD79A * (−1.926)] + [CSF3R *(−1.4990)] + [MTNR1B *(2.3731)] + [NPPC * (1.1894)]. The AUC of the PI in predicting the prognosis of OS as analyzed using the ROC curve was 0.761 (Figure 5(a)), showing that the PI signature had a better ability to predict survival. The results from both the UCR and MCR analyses are outlined in Figure 4(e and f). For the univariate risk score, metastases were found to be independent predictors.

### Predictive nomogram model of independent prognostic factors

A nomogram was established using the independent significant prognostic factors, which contain metastasis and risk score for predicting survival probability for twelve, twenty-four, and forty-eight months. The total points of this nomogram were summed and subsequently converted to reveal the probability of 1-year, 2-year and 3-year survival (Figure 5(b)). These results demonstrated that the nomogram displayed the highest levels of accuracy in predicting the survival of OS patients.

### GSEA

**Figure 6.**
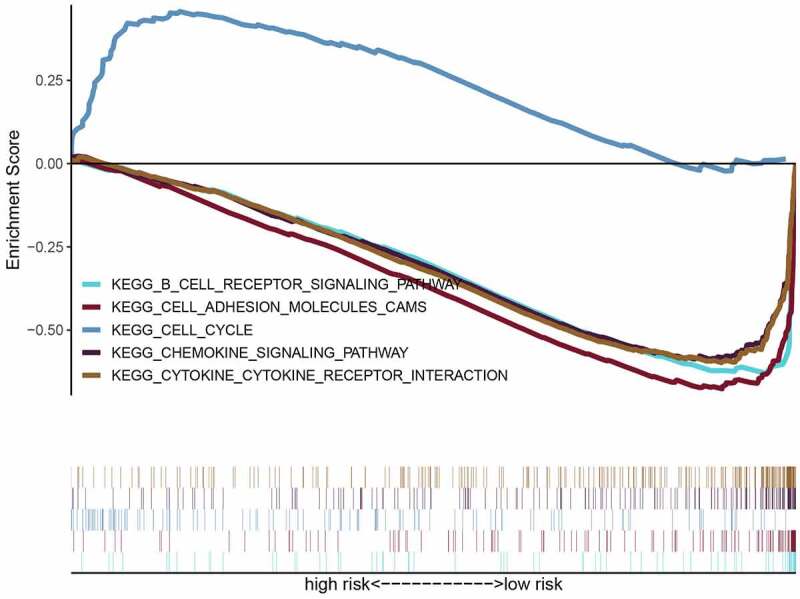
Enrichment plots of gene ontology annotation between high- and low-risk groups

To further research the underlying signaling pathway, GSEA was used to determine the associated signaling pathways between the two risk groups. The results showed that the high-risk groups were positively associated with the cell cycle and negatively related to chemokine and B cell receptor signaling pathways, cell adhesion molecules (CAMs), and cytokine-cytokine receptor (CCR) interactions (Figure 6 and Table 2). These results establish the basis for the follow-up OS immunotherapy.

**Table 2.** Immune-related gene sets that associated with high-risk group

NAME	ES	NES	NOM p-val	FDR q-val
Cell cycle	0.457	1.884	0.012	0.227
Cell adhesion molecules	−0.682	−2.302	0.000	0.002
B cell receptor signaling pathway	−0.645	−2.275	0.000	0.002
Chemokine Signaling Pathway	−0.594	−2.265	0.000	0.002
Cytokine cytokine receptor interaction	−0.601	−2.225	0.000	0.003

ES, enrichment score; NES, normalized enrichment score; NOM, nominal; FDR, false discovery rate.

### Survival risk is related to the proportion of the various TIIC types in OS

**Figure 7.**
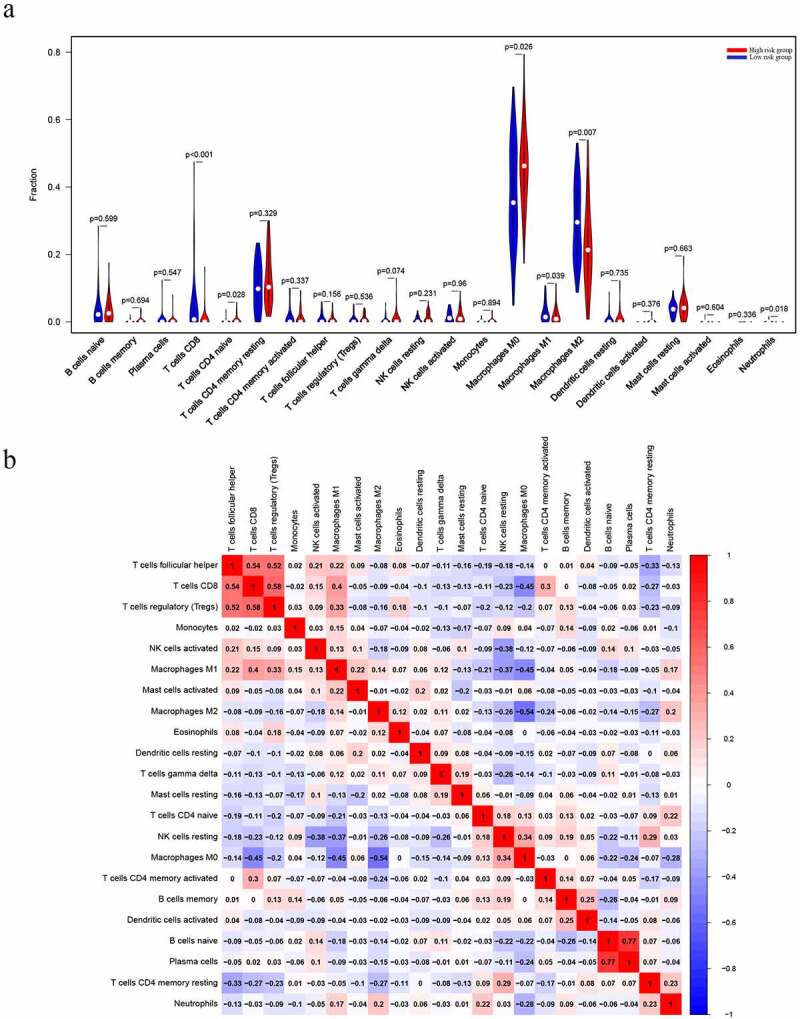
Evaluation of the proportions of TIICs based on CIBERSORT. (a) The varied proportions of 22 subtypes of immune cells in tumor samples from the high- and low-risk groups. (b) Heatmap of 22 immune-infiltrating cell types in tumor samples

**Figure 8.**
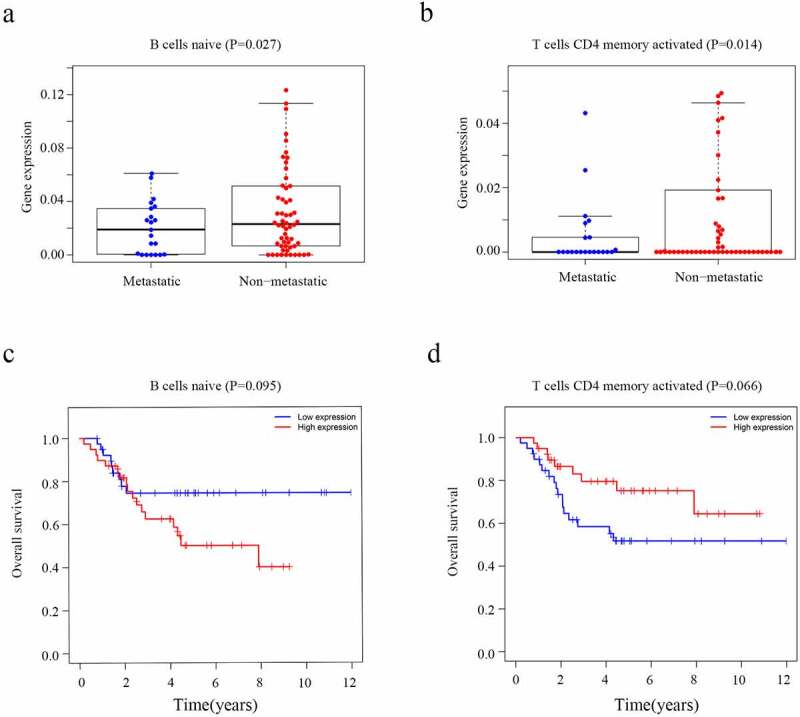
The level of immune cells and Kaplan-Meier analysis in patients with OS. (a) Relationship between the level of CD4 naïve T cells and metastasis. (b) Relationship between the level of T cells CD4 memory activated cells and metastasis. (c) The association between CD4 naïve T cells and overall survival of OS. (d) The association between T cells CD4 memory activated and overall survival of OS

The CIBERSORT algorithm was used to calculate the fraction of the immune cells and thus further identify the differences in the TIIC profiles between the two risk groups. High-risk patients exhibited a higher level of CD4 naïve T cells (P = 0.028) and M0 macrophages (P = 0.026) (Figure 7(a)). In contrast, the low-risk group had higher fractions of CD8 T cells (P < 0.001), M2 macrophages (P = 0.007) and neutrophils (P = 0.018) (Figure 7(a)). Furthermore, higher level of CD4 naïve T cells (P = 0.027) and T cells CD4 memory activated (P = 0.014) nagative associated with metastasis of osteosarcoma (Figure 8(a, b)). However, the level of CD4 naïve T cells (P = 0.095) and T cells CD4 memory activated (P = 0.066) which were not related with overall survival of osteosarcoma patients (Figure 8(c, d)). We believe that this may be related to the small sample size. It is necessary to further expand the sample size for research in the future.

## Discussion

OS is one of the most prevalent bone malignancies in adolescents and young adults. The slow progress of molecular targeted therapy and the lack of effective prognostic biomarkers are issues for OS patients. It is necessary to understand the mechanism underlying this type of cancer [18,19]. Therefore, the exploration of new immune factors may offer novel molecular targets in this tumor therapy method. However, most existing studies were conducted on single genes or proteins. To identify key immunity genes for OS, we screened DIRGs, and four key prognostic IRGs were identified, all of which may play a crucial role in OS and could be potential therapeutic targets.

In the current study, we looked at the IRG expression profile of samples from the TARGET database and aimed to identify prognostic biomarkers for patients with OS. We first screened 38 DIRGs between nonmetastatic samples and metastatic samples. Considering that these DIRGs may be associated with tumorigenesis and the development of OS, GO and KEGG enrichment analyses of these DIRGs were also performed in DAVID datasets. Interestingly, functional analysis and KEGG pathway analysis revealed that the DIRGs were enriched in immune-associated functions and pathways, including lymphocyte proliferation, leukocyte migration and T cell activation. In the univariate survival analysis, the expression of nine IRGs was found to be markedly related to the TARGET database prognosis. The PI model was then constructed by multivariate Cox regression based on four key prognostic IRGs (CD79A, CSF3R, MTNR1B and NPPC), and this model acted as an independent prognostic indicator of survival in OS patients. Most of these genes were closely related to the development, progression and prognosis of tumors in previous studies. GSEA demonstrated significant enrichment of the 4-gene signature associated with chemokine and B cell receptor signaling pathways, CCR interaction, and CAMs.

CD79a is part of the B cell receptor, and most T cell neoplasms do not express CD79a proteins [20]. High immune infiltration of CD79a+ B cells in the tumor stroma tends to be associated with good survival in colorectal liver metastasis patients [21]. However, little is known regarding the underlying regulatory mechanism of CD79a in OS growth and metastasis. CSF3R hypermethylation is related to cisplatin resistance in hepatoblastoma patients [22]. CSF3R expression is positively related to prognosis in patients with acute myeloid leukemia [23]. In this study, we found that the low-risk group had significantly higher CD79A and CSF3R expression than the high-risk group, which suggests that tumor immunity might be related to somatic mutations. MTNR1B (melatonin receptor 1B) is the membrane receptor of melatonin. The tumor-suppressive role of MTNR1B has been studied in numerous tumors (β-catenin signaling), including prostate, lung, gastrointestinal, and breast cancers [24,25]. However, the roles of NPPC in tumors remain unclear and need further study.

Immune infiltration is a feature of most cancers, and many cancers have a complex chemokine network that modulates tumor cell growth, survival and migration, as well as the extent and phenotype of this infiltration [26,27]. Neutrophils might play an essential role in tumor progression and development by providing a suitable microenvironment for their growth, thus serving as biomarkers for inflammation and as therapeutic targets [28,29]. On the other hand, tumor-associated macrophages (TAMs) promote tumor progression by providing nutrients for the invasion of tumor cells [30,31]. In this study, we found that the high-risk group had higher fractions of CD4 naïve T cells and M0 macrophages. However, there were higher fractions of CD8 T cells, M2 macrophages and neutrophils in the low-risk group. These data indicated that high risk group can affect immune cell infiltration signatures.

In summary, we developed a 4-gene-based risk model for OS prognosis prediction via a thorough analysis of IRGs. The model could provide new insights into the development of immunotherapies for OS. Moreover, the high-risk group closely correlated with several TIICs, particularly CD8 T cells, M2 macrophages, and CD4 naïve T cells. Moreover, chemokine and B cell receptor signaling pathways, CAMs, and CCR interactions were significantly enriched in the high-risk group of OS patients. In summary, a 4-gene-based risk model may serve as a marker for predicting prognosis and correlates with immune infiltration in OS. However, prospective studies are needed to confirm our findings to aid in personalized clinical practice.

## Conclusion

We developed a four-immune gene prognostic index of osteosarcoma, including CD79A, CSF3R, MTNR1B and NPPC. This prognostic index could be used as an instrumental variable in the prognosis prediction of osteosarcoma. The underlying molecular mechanisms may affect immune-related biological processes and TIICs. In conclusion, prognostic index could predict prognosis for patients with OS and might provide novel insights into the relationship between OS and tumor immune infiltration.

## Supplementary Material

Supplemental Material

## Data Availability

All analyzed data are included in this published article and its supplementary information file. The original data are available upon reasonable request to the corresponding author.

## Highlights

1. An immune-related prognostic index to predict the prognosis of OS was constructed.
2. The prognostic index is an independent index for OS prognosis.
3. The prognostic index may affect immune-related biological processes and TIICs.
