# Supplementary figures and images for: Development of an immune-related prognostic index associated with osteosarcoma
Source: Bioengineered. 2020 Dec 29;12(1):172–82. doi: 10.1080/21655979.2020.1864096 (PMC8806312; doi:10.1080/21655979.2020.1864096)

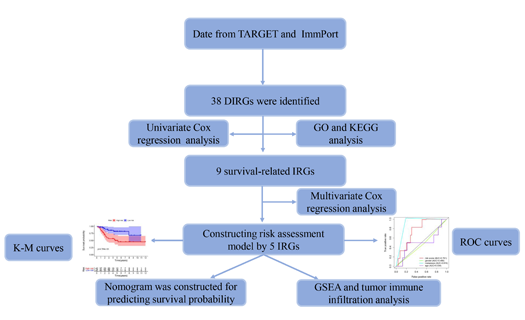

Supplement: Supplemental Material [file KBIE_A_1864096_SM2394.tif]
